# Some Results of Fermatean Fuzzy Set on Subalgebras and Ideals of Bn-Algebras

**DOI:** 10.12688/f1000research.175837.2

**Published:** 2026-05-07

**Authors:** Derebew Derso, Gerima Tefera, Eshetu Assen Teshome

**Affiliations:** 1 Mathematics, Woldia University, Woldia, Amhara, 400, Ethiopia; 2 Mathematics, Wollo University, Dessie, Amhara, Ethiopia

**Keywords:** Fermatean fuzzy set, level set, fermatean subalgebra, Fermatean fuzzy subalgebra, Fermatean ideal, Fermatean homomorphism

## Abstract

**Background:**

Classical fuzzy sets have been generalized to better model uncertainty, leading to developments such as intuitionistic, Pythagorean, and Fermatean fuzzy sets. Fermatean fuzzy sets (ffss), characterized by the cube sum constraint on membership and non-membership degrees, provide a more flexible framework for handling complex uncertainty. BN-algebras are important algebraic structures with applications in logic and information theory.

**Methods:**

This study integrates Fermatean fuzzy sets with BN-algebras by introducing the notions of Fermatean fuzzy subalgebras and Fermatean fuzzy ideals. Level cuts of Fermatean fuzzy sets are defined and analyzed, and algebraic techniques are employed to investigate their structural properties.

**Results:**

It is shown that Fermatean fuzzy level subalgebras and Fermatean fuzzy level ideals correspond to classical subalgebras and ideals of BN-algebras. Several characterizations and closure properties are established, supported by illustrative examples and rigorous proofs.

**Conclusions:**

The results demonstrate that Fermatean fuzzy sets significantly enrich the theory of BN-algebras by accommodating higher degrees of uncertainty. This framework provides a solid theoretical foundation for further studies and potential applications in algebraic logic and uncertainty-based systems.

## 1. Introduction

The concept of fuzzy sets (fs), introduced by zadeh, ^13^ and has revolutionized the handling of uncertainty by allowing elements to have varying degrees of membership. this foundational idea was extended by atanassov ^3^ with intuitionistic fuzzy sets (IFS), which added a degree of non-membership and yager ^12^ further advanced this field with pythagorean fuzzy sets (PFS), where the square sum of membership and non-membership degrees is less than or equal to one. Adak, a.k., nilkaumal, and bb arman ^1^ introduced Fermatean fuzzy semi-prime order semi groups, and the concept of Fermatean fuzzy set (FFS) was naturalized by senapati.t and yager, ^10^ anas and et al. ^2^ introduced the concepts of the direct product of sets that address the importance of Fermatean neutrosophic elements. y., komori ^7^ introduced varieties of BCC-Algebra, the relation other algebraic structures, and j.neggers and H.S.kim ^8^ introduced B-algebras, which are related to several generalizations of BCK-algebras, such as BCIci-algebras, BCH-algebras, BCC-algebras, BH-algebras, and ^9^ D-algebras. In addition, Kim, C.B., and Km, hH.s discussed the concepts of BN-algebras with different structures, and grzegorz dymek and andrzej walendziak extend the concepts of ideals of BN-algebra to fuzzy ideals of BN-algebra ^4^ with different properties. In this paper, we initiate the concept of a Fermatean fuzzy set on the ideals of BN-algebras and study its application. We state and prove some theorems discussed in the Fermatean fuzzy set on the ideals of BN-algebras and applications. We also extend the notions of an ideal and a normal ideal in a Fermatean fuzzy set on the ideals of BN-algebras.

## 2. Preliminaries

Definition 2.1: ^3^Let X be a universe of study. An intuitionistic fuzzy sets (IFSs) in X is an object

$\text{IFS}=\left\{\left(x,\delta_{\text{IFS}}\left(x\right),\theta_{\text{IFS}}\left(x\right)\right),\;x\;\in \;X\right\}$ , where $\delta_{\text{IFS}}:X\to \left[0,1\right]\;$ and $\theta_{\text{IFS}}:X\to \left[0,1\right],\theta_{\text{IFS}}\left(x\right)=1-\delta_{\text{IFS}}\left(x\right)$ satisfy the following criteria:

$0\;\leq \;\delta_{\text{IFS}}\left(x\right)+\theta_{\text{IFS}}\left(x\right)\;\leq \;1$ , for all x in *X*, where $\delta_{\text{IFS}}\left(x\right)$ and $\theta_{\text{IFS}}\left(x\right)$ represents degree membership value and non-degree membership value of an element in IFS.

Definition 2.2: ^12^The pythagorean fuzzy set defined on a nonempty set X is of the form

$$P=\left\{\left(x,\theta_{P}\left(x\right),\delta_{P}\left(x\right)\right)\;|\;x\;\in \;X\right\},$$

where $\theta_{P}\left(x\right)$ and $\delta_{P}\left(x\right)$ are the degree membership and non-degree membership functions from X to [0, 1] and

$0\;\leq \;{\left(\theta_{P}\left(x\right)\right)}^{2}+{\left(\delta_{P}\left(x\right)\right)}^{2}\;\leq \;1$ for all x∈X.

Definition 2.3: ^10^Let X be a universal set. A Fermatean fuzzy set (FF) in X is

$$F=\left\{\left(x,\theta_{F}\left(x\right),\delta_{F}\left(x\right)\right)\;|\;x\;\in \;X\right\}$$

with $0\;\leq \;{\left(\theta_{F}\left(x\right)\right)}^{3}+{\left(\delta_{F}\left(x\right)\right)}^{3}\;\leq \;1$ for all x∈X, where $\theta_{F}:X\to \left[0,1\right]$ and $\delta_{F}:X\to \left[0,1\right]$ represent the degree membership and non-degree membership functions, respectively.

Definition 2.4: ^1^Let X be a universe of study. A Fermatean fuzzy set F in X is an object

$$F=\left\{\left(x,\delta_{F}\left(x\right),\theta_{F}\left(x\right)\right)\;|\;x\;\in \;X\right\},$$

where $\delta_{F}:X\to \left[0,1\right]$ and $\theta_{F}:X\to \left[0,1\right],\theta_{F}\left(x\right)=1-\delta_{F}\left(x\right)$ satisfy the following criteria:

$$0\;\leq \;\delta_{F}{\left(x\right)}^{3}+\theta_{F}{\left(x\right)}^{3}\;\leq \;1\;\text{for}\;\mathrm{all}\;x\;\in \;X,$$

where $\delta_{F}\left(x\right)$ and $\theta_{F}\left(x\right)\;$ represent degree membership value and non-degree membership value of an element in F .

Definition 2.5: ^12^Algebra (X,∗,0) of type (2, 0) is called a BN-algebra if for all x,y,z∈X the following identities hold:

1.
x∗x=0

2.
x∗0=x

3.
(x∗y)∗z=(0∗z)∗(y∗x). Let (X,∗,0) be a BN-algebra. We define binary relation ≤ on X by x≤y if and only if x∗y=0 . for any x∈X , if x≤0 , then x=0.

Example 2.6: Let ℝ be the set of real numbers and let r = (ℝ, *, 0) be the algebra with the operation * defined by

x∗y={xify=0yifx=00,otherwise

then r is a BN-algebra.

Proposition 2.7: ^5^If (X,∗,0) is a BN-algebra then for all x,y∈X

1.
0∗(0∗x)=x

2.
0∗(x∗y)=y∗x

3.
y∗x=(0∗x)∗(0∗y)

4.
x∗y=0⇒y∗x=0

5.
0∗x=0∗y⇒x=y.

Definition 2.8: ^6^If BCI-algebra satisfies the condition 0∗x=x it becomes a BCK-algebra. An algebra (X,∗,0) satisfies the condition:

1.
x∗x=0

2.
x∗0=x

3.
(x∗y)∗z=x∗(z∗(0∗y)) for all x,y∈X as a generalization of BCK-algebra.

Definition 2.9: ^7^Algebra (X,∗,0) is said to be a BM-algebra if it satisfies the following axioms for all x,y,z∈X:

1.
x∗0=x

2.
(z∗x)∗(z∗y)=y∗x

Definition 2.10: ^11^Algebra (X,∗,0) of type (2, 0) is called a BF-algebra if it satisfies the following axioms for all x,y∈X:

1.
x∗x=0

2.
x∗0=x

3.
0∗(x∗y)=y∗x

## 3. Main results

### 3.1 Fermatean BN-Sub algebra of BN-Algebras

Definition 3.1: Let *F* be a Fermatean fuzzy set (FFS) in X . Then *F* is called a Fermatean fuzzy BN-subalgebra of *x* if the following conditions hold for all x,y∈X:

1.
$\delta_{F}\left(x*y\right)\;\geq \;\delta_{F}\left(x\right)\;\wedge \;\delta_{F}\left(y\right)$

2.
$\theta_{F}\left(x*y\right)\;\leq \;\theta_{F}\left(x\right)\;\vee \;\theta_{F}\left(y\right)$

where $\theta_{F}\left(x\right)=1-\delta_{F}\left(x\right),\delta_{F}:X\to \left[0,1\right],\theta_{F}:X\to \left[0,1\right]$ for all x∈X , and

$$0\;\leq \;{\left(\delta_{F}\left(x\right)\right)}^{3}+{\left(\theta_{F}\left(x\right)\right)}^{3}\;\leq \;1.$$

Example 3.2: Let *x* = {0, 1, 2, 3, 4} be a set, and let * be defined by Table 1. The operation table defining the BN-algebra structure used in the following example is given in Table 1.

Then (X,∗,0) is a BN-algebra. Define $\delta_{F}:X\to \left[0,1\right]$ by

$$\delta_{F}\;\left(0\right)=0.9,\;\delta_{F}\left(1\right)=0.5=\delta_{F}\left(3\right),\;\delta_{F}\left(4\right)=0.6=\delta_{F}\left(2\right).$$

Define $\theta_{F}\left(x\right)=1-\delta_{F}\left(x\right).\;\text{Then}\;\theta_{F}\left(0\right)=0.1,\;\theta_{F}\left(1\right)=0.5,\;\theta_{F}\left(2\right)=0.4,\;\theta_{F}\;\left(3\right)=0.5,\;\theta_{F}\left(4\right)=0.4.$

Let S={0,1}⊆X . Then S is a subalgebra of the BN-algebra X .

We have $\delta_{F}\left(0*1\right)=\delta_{F}\left(1\right)\;\geq \;0.9\;\wedge \;0.5=\delta_{F}\left(0\right)\;\wedge \;\delta_{F}\left(1\right)$ implies $\delta_{F}\left(0*1\right)\;\geq \;\delta_{F}\;\left(0\right)\;\wedge \;\delta_{F}\left(1\right).$

Also $\theta_{F}\left(0*1\right)=\theta_{F}\left(1\right)\;\leq \;0.1\;\vee \;0.5=\theta_{F}\left(0\right)\;\vee \;\theta_{F}\left(1\right).$

Hence $\delta_{F}\left(x*y\right)\;\geq \;\delta_{F}\left(x\right)\;\wedge \;\delta_{F}\left(y\right)$ and $\theta_{F}\left(x*y\right)\;\leq \;\theta_{F}\left(x\right)\;\vee \;\theta_{F}\left(y\right)$ for all x,y∈X.

Thus, $F=\left\{x\;\in \;S:\delta_{F}\left(x\right),\theta_{F}\left(x\right)\right\}$ is a Fermatean fuzzy subalgebra of X.

**Table 1.** Fermatean fuzzy sub algebra.

*	0	1	2	3	4
0	0	1	2	2	4
1	1	0	3	2	3
2	2	4	0	1	2
3	3	3	2	0	0
4	4	2	3	3	0

Proposition 3.3: Let *X* be a BN-algebra and s be a subalgebra of x. Then

$$F=\left\{x\;\in \;S:\theta_{F}\left(x\right)=0\;\&\;\delta_{F}\left(x\right)=1\right\}$$

is a Fermatean fuzzy subalgebra of *x* with

$$0\;\leq \;{\left(\theta_{F}\left(x\right)\right)}^{3}+{\left(\delta_{F}\left(x\right)\right)}^{3}\;\leq \;1,\theta_{F}:X\to \left[0,1\right],\delta_{F}:X\to \left[0,1\right]$$

and $\theta_{F}\left(x\right)=1-\delta_{F}\left(x\right)$ for x∈X .

Proof: Let S be a subalgebra of X . Then x,y∈S imply x∗y∈S . Since $\theta_{F}\left(0\right)=0$ and $\delta_{F}\left(0\right)=$ 1, it follows that 0∈F . Hence F is nonempty.

Let x,y∈F . Then $\theta_{F}\left(x\right)=0,\delta_{F}\left(x\right)=1\;$ and $\;\theta_{F}\left(y\right)=0,\delta_{F}\left(y\right)=1$ . Since x,y∈F implies x,y∈S , it follows that x∗y∈S . We get $\theta_{F}\left(x\;*\;y\right)=0,\delta_{F}\left(x\;*\;y\right)=1.$

1.
$1=\delta_{F}\left(x\;*\;y\right)\;\geq \;1\;\wedge \;1=\delta_{F}\left(x\right)\;\wedge \;\delta_{F}\left(y\right)$ . It follows $\delta_{F}\left(x\;*\;y\right)\;\geq \;\delta_{F}\left(x\right)\;\wedge \;\delta_{F}\left(y\right)$ for all x,y∈S.

2.
$0=\theta_{F}\left(x\;*\;y\right)\;\leq \;0\;\vee \;0=\theta_{F}\left(x\right)\;\vee \;\theta_{F}\left(y\right).$ We get $0=\theta_{F}\left(x\;*\;y\right)\;\leq \;\theta_{F}\left(x\right)\;\vee \;\theta_{F}\left(y\right)$ for all x,y∈S.

Hence F is a Fermatean fuzzy subalgebra of x. Moreover $0\;\leq \;{\left(\theta_{F}\left(x\right)\right)}^{3}+{\left(\delta_{F}\left(x\right)\right)}^{3}\;\leq \;1\;$ for all x,y∈F.

Definition 3.4: Let X be a BN-algebra and $S_{\mathrm{FF}}\left(X\right)$ be the set of all Fermatean fuzzy subalgebras of X . Then for $\delta_{F},\theta_{F}\;\in \;S_{\mathrm{FF}}\left(X\right)$ we have:

1.
$U\left(\delta_{F},t\right)={\left(\delta_{F}\right)}_{t}=\left\{x\;\in \;S:\delta_{F}\left(x\right)\;\geq \;t,t\;\in \;\left[0,1\right]\right\}$

2.
$L\left(\theta_{F},k\right)={\left(\theta_{F}\right)}_{k}=\left\{x\;\in \;S:\theta_{F}\left(x\right)\;\leq \;k,k\;\in \;\left[0,1\right]\right\}$

are called level cuts of s. Here $U\left(\delta_{F},t\right)$ and $L\left(\theta_{F},k\right)$ are called the upper-level cut and lower-level cut of s respectively.

Theorem 3.5:
$F=\left\{x\;\in \;X,\delta_{F}\left(x\right),\theta_{F}\left(x\right)\right\}$ is a Fermatean subalgebra of *x* if and only if $U\left(\delta_{F},t\right)$ and $L\left(\theta_{F},k\right)$ for k,t∈[0,1] are subalgebra of X .

Proof: Assume f is a Fermatean fuzzy subalgebra of x. We need to prove $U\left(\delta_{F},t\right)$ and $L\left(\theta_{F},k\right)$ are subalgebra of X for k,t∈[0,1].

Let $x_{0}\;\in \;X$ such that $\delta_{F}\left(x_{0}\right)\;\geq \;t.$ Since

$$\;\delta_{F}\left(0\right)=\delta_{F}\left(x_{0}*x_{0}\right)=\delta_{F}\left(x_{0}\right)\;\wedge \;\delta_{F}\left(x_{0}\right)=\delta_{F}$$

$\left(x_{0}\right)\;\geq \;t$ , implies $\delta_{F}\left(0\right)\;\geq \;t\;$ and hence $0\;\in \;U\left(\delta_{F},t\right).$ Therefore, $U\left(\delta_{F},t\right)$ is nonempty.

Let $x,y\;\in \;U\left(\delta_{F},t\right).$ Then $\delta_{F}\left(x\right)\;\geq \;t\;$ and $\delta_{F}\left(y\right)\;\geq \;t$ . Put $\delta_{F}\left(x\right)=t$ and $\;\delta_{F}\left(y\right)=t$ . Now $\delta_{F}\left(x\;*\;y\right)\;\geq \;\delta_{F}\left(x\right)\;\wedge \;\delta_{F}\left(y\right)=t\;\wedge \;t=t.$ It follows that $\delta_{F}\left(x\;*\;y\right)\;\geq \;t.$ Hence $x\;*\;y\;\in \;U\left(\delta_{F},t\right),$ which implies $U\left(\delta_{F},t\right)$ is a subalgebra of X . Similar result holds for $0<t_{1}<t_{2}\;\leq \;1$ or $0<t_{2}<t_{1}\;\leq \;1$ .

Again let $x_{0}\;\in \;X$ such that $\;\theta_{F}\left(x\_0\right)\;\leq \;k$ , for $\delta_{F}\left(x\_0\right)=1-\theta_{F}\left(x\_0\right),k\;\in \;\left[0,1\right].\;\theta_{F}\left(0\right)=\theta_{F}\left(x_{0}\;*\;x_{0}\right)\;\leq \;\theta_{F}\left(x\_0\right)\;\vee \;\theta_{F}\left(x\_0\right)=\theta_{F}\left(x\_0\right)\;\leq \;k.$ Imply that $0\;\in \;L\left(\theta_{F},k\right)$ which implies $L\left(\theta_{F},k\right)$ is non-empty.

Let $x,y\;\in \;L\left(\theta_{F},k\right)$ then $\theta_{F}\left(x\right)\;\leq \;k$ and $\;\theta_{F}\left(y\right)\;\leq \;k$ . Put $\theta_{F}\left(x\right)=k=\theta_{F}\left(y\right)$ now $\theta_{F}\left(x\;*\;y\right)\;\leq \;\theta_{F}\left(x\right)\;\vee \;\theta_{F}\left(y\right)=k\;\vee \;k=k$ which imply $\;\theta_{F}\left(x\;*\;y\right)\;\leq \;k$ we get $x\;*\;y\;\in \;L\left(\theta_{F},k\right)$ therefore $L\left(\theta_{F},k\right)$ is a subalgebra of x a similar result holds for $0<k_{1}<k_{2}\;\leq \;1\;\text{or}\;0<k_{2}<k_{1}\;\leq \;1.$

Conversely, assume $\;U(\delta_{F},t$ ) and $L\left(\theta_{F},k\right)$ are subalgebras of X . We must prove $F=\left\{x\;\in \;X,\delta_{F}\left(x\right),\theta_{F}\left(x\right)\right\}$ is a Fermatean fuzzy subalgebra of X .

Let t,k∈[0,1] and because $U\left(\delta_{F},t\right)$ and $L\left(\theta_{F},k\right)$ are subalgebras, we have $U\left(\delta_{F},t\right)$ and $L\left(\theta_{F},k\right)\;$ nonempty. Let $x_{0}\;\in \;U\left(\delta_{F},t\right)$ and $x_{0}\;\in \;L\left(\theta_{F},k\right)$ then $\delta_{F}\left(x_{0}\right)\;\geq \;t$ and $\;\theta_{F}\left(x_{0}\right)\;\leq \;k$ .

Suppose $\delta_{F}\left(x\;*\;y\right)<\delta_{F}\left(x\right)\;\wedge \;\delta_{F}\left(y\right)$ then put $\;\beta =½\left\{\delta_{F}\left(x\;*\;y\right)+\delta_{F}\left(x\right)\;\wedge \;\delta_{F}\left(y\right)\right\}$ so that $\delta_{F}\left(x\;*\;y\right)<\beta <\delta_{F}\left(x\right)\;\wedge \;\delta_{F}\left(y\right)\;\leq \;\delta_{F}\left(x\;*\;y\right)$ implies that $\delta_{F}\left(x\;*\;y\right)<\delta_{F}\left(x\;*\;y\right)\;$ which is a contradiction. Hence $\delta_{F}\left(x\;*\;y\right)\;\geq \;\delta_{F}\left(x\right)\;\wedge \;\delta_{F}\left(y\right)\;$ for x,y∈X the cases $x_{0}\;\in \;U\left(\delta_{F},t\right)\;$ and $y_{0}\;\in \;L\left(\theta_{F},k\right)$ with $x_{0}\neq y_{0}$ followed by a similar argument.

Again, let x,y∈X such that $\theta_{F}\left(x\;*\;y\right)>\theta_{F}\left(x\right)\;\vee \;\theta_{F}\left(y\right)$ . Then put $\gamma =½\left\{\theta_{F}\left(x\;*\;y\right)+\theta_{F}\left(x\right)\;\vee \;\theta_{F}\left(y\right)\right\}$ then we have $\theta_{F}\left(x\;*\;y\right)>\gamma >\theta_{F}\left(x\right)\;\vee \;\theta_{F}\left(y\right)\;\geq \;\theta_{F}\left(x\;*\;y\right)$ which implies that $\theta_{F}\left(x\;*\;y\right)>\theta_{F}\left(x\;*\;y\right)$ which is not correct. Hence $\theta_{F}\left(x\;*\;y\right)\;\leq \;\theta_{F}\left(x\right)\;\vee \;\theta_{F}\left(y\right)$ for all x,y∈X the cases $x_{0}\;\in \;U\left(\delta_{F},t\right)$ and $y_{0}\;\in \;L\left(\theta_{F},k\right)$ with $x_{0}\neq y_{0}$ follow by a similar argument thus $F=\left\{x\;\in \;X,\delta_{F}\left(x\right),\theta_{F}\left(x\right)\right\}$ is a Fermatean fuzzy subalgebra of X.

Definition 3.6: Let X be a BN-algebra and let s be a subalgebra of X . Then

$$F=\left\{x\;\in \;S:\left(x,\delta_{F}\left(x\right),\theta_{F}\left(x\right)\right)\right\}$$

where $\;\theta_{F}=1-\delta_{F},\delta_{F}:X\to \left[0,1\right],\theta_{F}:X\to \left[0,1\right]$ . Then f is a Fermatean fuzzy subalgebra of *x* if x,y∈F⇒x∗y∈F .

Proposition 3.7: Let *x* be a BN-algebra and let s be a subalgebra of x then

$$F=\left\{x\;\in \;S:\theta_{F}\left(x\right)=0\;\;\&\;\delta_{F}\left(x\right)=1\right\}$$

Is a Fermatean fuzzy subalgebra of X with $0\;\leq \;{\left(\theta_{F}\left(x\right)\right)}^{3}+{\left(\delta_{F}\left(x\right)\right)}^{3}\;\leq \;1,\;\theta_{F}:X\to \left[0,1\right],\;\delta_{F}:X\to \left[0,1\right]$ and $\theta_{F}\left(x\right)=1-\delta_{F}\left(x\right)\;$ for x∈X.

Proof: Let *S* be a subalgebra of X then x,y∈S imply x∗y∈S since $\theta_{F}\left(0\right)=0$ and $\;\delta_{F}\left(0\right)=1$ , it follows that 0∈F hence F is nonempty.

Let x,y∈F then $\theta_{F}\left(x\right)=0$ and $\delta_{F}\left(x\right)=1$ and $\theta_{F}\left(y\right)=0$ and $\;\delta_{F}\left(y\right)=1$ since x,y∈F imply x,y∈S , it follows that x∗y∈S we get $\theta_{F}\left(x\;*\;y\right)=0,\delta_{F}\left(x\;*\;y\right)=1.$

1.
$0=\theta_{F}\left(x\;*\;y\right)\;\geq \;0\;\wedge \;0=\theta_{F}\left(x\right)\;\wedge \;\theta_{F}\left(y\right)$ it follows $\theta_{F}\left(x\;*\;y\right)\;\geq \;\theta_{F}\left(x\right)\;\wedge \;\theta_{F}\left(y\right)$ for all x,y∈S.

2.
$1=\delta_{F}\left(x\;*\;y\right)\;\leq \;1\;\vee \;1=\delta_{F}\left(x\right)\;\vee \;\delta_{F}\left(y\right)$ we get $\delta_{F}\left(x\;*\;y\right)\;\leq \;\delta_{F}\left(x\right)\;\vee \;\delta_{F}\left(y\right)$ for all x,y∈S.

Hence *F* is a Fermatean fuzzy subalgebra of x moreover $0\;\leq \;{\left(\theta_{F}\left(x\right)\right)}^{3}+{\left(\delta_{F}\left(x\right)\right)}^{3}\;\leq \;1$ for all x,y∈F .

Theorem 3.8: Let *S* be a subalgebra of X then ${\left(\delta_{F},\theta_{F}\right)}_{\left\{(t,k)\right\}}$ is a Fermatean level subset of s if and only if

$$F=\left\{x\;\in \;S:\delta_{F}\left(x\right)\;\geq \;t,\theta_{F}\left(x\right)\;\leq \;k,t,k\;\in \;\left[0,1\right]\right\}$$

Is a Fermatean fuzzy subalgebra of X here $0\;\leq \;{\left(\delta_{F}\left(x\right)\right)}^{3}+{\left(\theta_{F}\left(x\right)\right)}^{3}\;\leq \;1$ for all x∈S .

Proof: Let ${\left(\delta_{F},\theta_{F}\right)}_{\left\{(t,k)\right\}}$ be a level subset of S in X .

Claim: *F* is a Fermatean fuzzy subalgebra.

1. Since $0\;\in \;F,\delta_{F}\left(0\right)\;\geq \;t$ and $\;\theta_{F}\left(0\right)\;\leq \;k$ to show closure put $\delta_{F}\left(0\right)=0$ and $\theta_{F}\left(x\right)=0\Rightarrow F$ is nonempty.
2. Let x,y∈F then $\delta_{F}\left(x\right)\;\geq \;t,\theta_{F}\left(x\right)\;\leq \;k$ and $\delta_{F}\left(y\right)\;\geq \;t,\theta_{F}\left(y\right)\;\leq \;k$ put $\delta_{F}\left(x\right)=\delta_{F}\left(y\right)=t=t\;\wedge \;t=\inf\left\{t,t\right\}=\delta_{F}\left(x\right)\;\wedge \;\delta_{F}\left(y\right)\;\leq \;\delta_{F}\left(x\;*\;y\right)\Rightarrow \delta_{F}\left(x\;*\;y\right)\;\geq \;t\Rightarrow x\;*\;y\;\in \;F.$

3.
$\mathrm{Let}\;x,y\;\in \;F\Rightarrow \theta_{F}\left(x\right)\;\leq \;k\;$ and $\;\theta_{F}\left(y\right)\;\leq \;k.\;\mathrm{Put}\;k=\theta_{F}\left(x\right)=\theta_{F}\left(y\right).\;k=\sup\left\{k,k\right\}=\sup\left\{\theta_{F}\left(x\right),\theta_{F}\left(y\right)\right\}\;\geq \;\theta_{F}\left(x\;*\;y\right)\Rightarrow \theta_{F}\left(x\;*\;y\right)\;\leq \;k\Rightarrow x\;*\;y\;\in \;F.$

Hence *F* is a Fermatean fuzzy subalgebra of X.

### 3.2 Fermatean Fuzzy Ideal of BN-Algebra

Definition 3.9: Let *X* be a BN Algebra. Then Fermatean fuzzy ideal of *x* is defined by:

1.
$\delta_{F}\left(0\right)\;\geq \;\delta_{F}\left(x\right),x\;\in \;X.$

2.
$\delta_{F}\left(x\right)\;\geq \;\delta_{F}\left(x\;*\;y\right)\;\wedge \;\delta_{F}\left(y\right).$

3.
$\theta_{F}\left(x\right)\;\leq \;\theta_{F}\left(x\;*\;y\right)\;\vee \;\theta_{F}\left(y\right),\;\text{for}\;\mathrm{all}\;x,y\;\in \;X$ .

where $\delta_{F}:X\to \left[0,1\right]\;$ and $\theta_{F}:X\to \left[0,1\right],\theta_{F}\left(x\right)=1-\delta_{F}\left(x\right)\;$ for all x∈X.

Example 3.10: Let *X* = {0, a, b, c} and * be defined by Table 2.

Let $0\;\leq \;t_{3}<t_{2}<t_{1}<1.$ Define a fuzzy subset $\delta_{F}:X\to \left[0,1\right]$ by

δF(x)={t1ifx=0t2ifx=bt3ifx∈{a,c}

and $\theta_{F}\left(x\right)=1-\delta_{F}\left(x\right).$ We must show $\delta_{F}$ and $\theta_{F}$ satisfy the condition of the Fermatean fuzzy ideal of X .

1. if x=b and y=a, we have $\delta_{F}\left(b\right)=t_{2}\;\geq \;\delta_{F}\left(b\;*\;a\right)\;\wedge \;\delta_{F}\left(a\right)$ hence it holds. $\delta_{F}\left(0\right)=t_{1}>t_{2}>t_{3}$ it follows that $\delta_{F}\left(0\right)\;\geq \;\delta_{F}\left(x\right)$ for all x∈X.

2.
$\theta_{F}\left(b\right)=1-t_{2}\;\leq \;\left(1-t_{2}\right)\;\vee \;\left(1-t_{3}\right)=\theta_{F}\left(b\right)\;\vee \;\theta_{F}\left(b\;*\;a\right).\;\text{Imply}\;\theta_{F}\left(b\right)=1-t_{2}\;\leq \;\theta_{F}\left(b\right)\;\vee \;\theta_{F}\left(b\;*\;a\right).$

If x=a , then $\;\theta_{F}\left(a\right)=1-t_{3}\;\leq \;\left(1-t_{3}\right)\;\vee \;\left(1-t_{1}\right)=\theta_{F}\left(a\right)\;\vee \;\theta_{F}\left(a\;*\;a\right)$ .

**Table 2.** Fermatean fuzzy ideal.

*	0	a	b	c
0	0	a	b	c
a	a	0	a	a
b	b	a	0	a
c	c	a	a	0

Proposition 3.11: For a Fermatean fuzzy ideal $I_{\mathrm{FF}}$ of *x* and any x,y∈X . if x≤y then

1.
$\delta_{F}\left(x\right)\;\leq \;\delta_{F}\left(y\right)$

2.
$\theta_{F}\left(y\right)\;\leq \;\theta_{F}\left(x\right)$

Proof: Let x,y∈X such that x≤y implies x∗y=0 . it follows y∗x=0.

1.
$\delta_{F}\left(y\right)\;\geq \;\delta_{F}\left(y\;*\;x\right)\;\wedge \;\delta_{F}\left(x\right)=\delta_{F}\left(0\right)\;\wedge \;\delta_{F}\left(x\right)=\delta_{F}\left(x\right).\;\text{Hence}\;\delta_{F}\left(y\right)\;\geq \;\delta_{F}\left(x\right).$

2.
$\theta_{F}\left(y\right)=1-\delta_{F}\left(y\right)\;\leq \;\left(1-\delta_{F}\left(y\;*\;x\right)\right)\;\vee \;\left(1-\delta_{F}\left(x\right)\right)=\left(1-\delta_{F}\left(0\right)\right)\;\vee \;\left(1-\delta_{F}\left(x\right)\right)\;\leq \;1-\delta_{F}\left(x\right)=\theta_{F}\left(x\right).$ Hence $\theta_{F}\left(y\right)\;\leq \;\theta_{F}\left(x\right)$ for x≤y and x,y∈X.

Proposition 3.12: Let *I _FF_* be a Fermatean fuzzy ideal of a BN-algebra x then $\theta_{F}\left(0\right)\;\leq \;\theta_{F}\left(x\right)\;\text{for}\;\mathrm{all}\;x\;\in \;X,\delta_{F}:X\to \left[0,1\right]\;\text{and}\;\theta_{F}\left(x\right)=1-\delta_{F}\left(x\right)$ with the property $0\;\leq \;{\left(\theta_{F}\left(x\right)\right)}^{3}+{\left(\delta_{F}\left(x\right)\right)}^{3}\;\leq \;1.$

Proof: Let *I _FF_* be a Fermatean fuzzy ideal of a BN-algebra X and $\delta_{F}:X\to \left[0,1\right]$ be a degree membership function and $\theta_{F}\left(x\right)=1-\delta_{F}\left(x\right)$ for all x∈X.

$\theta_{F}\left(0\right)=1-\delta_{F}\left(0\right)\;\leq \;1-\delta_{F}\left(x\right)=\theta_{F}\left(x\right)$ for all x∈X hence $\theta_{F}\left(0\right)\;\leq \;\theta_{F}\left(x\right)$ for all x∈X.

Proposition 3.13: Let $I_{\mathrm{FF}}$ be a Fermatean fuzzy ideal of X and $J=I_{\mathrm{FF}}$ then

χJ(x)={1ifx∈J0ifx∉J.

then $\chi_{J}$ is a fuzzy ideal of X .

Proof: Let *j* be a Fermatean fuzzy ideal of *x* and

χJ(x)={1ifx∈J0ifx∉J

1. Let $\chi_{J}=\delta_{F}$ then we have $\delta_{F}\left(x\right)=1\;\geq \;\delta_{F}\left(x\right)\;$ for all x∈J and 0∈J but $\delta_{J}\left(x\right)=1$ it follows that $\;0\;\in \;\chi_{J}$ . $\delta_{F}\left(0\right)=\delta_{F}\left(x\right)$ for all $x\;\in \;\chi_{J}$ it follows that $\delta_{F}\left(0\right)\;\geq \;\delta_{F}\left(x\right)\;$ for all $x\;\in \;\chi_{J}.$

2. Let x∗y∈J and y∈J then $\delta_{F}\left(x\;*\;y\right)=1$ and $\delta_{F}\left(y\right)=1$ we get $\delta_{F\left(x\right)}\;\geq \;\delta_{F}\left(x\;*\;y\right)\;\wedge \;\delta_{F}\left(y\right)=1\;\wedge \;1=1$ imply $\delta_{F}\left(x\right)\;\geq \;1$ but $\delta_{F}\left(x\right)\;\leq \;1$ so that $\delta_{F}\left(x\right)=1$ hence $x\;\in \;\chi_{J}$ and x∈J consequently $\delta_{F}\left(x\right)\;\geq \;\delta_{F}\left(x\;*\;y\right)\;\wedge \;\delta_{F}\left(y\right)$ for x,y∈J therefore $\chi_{J}$ is a fuzzy ideal of X .

### 3.3 Fermatean level ideal of BN-Algebra

Definition 3.14: Let X be a BN-algebra and let $I_{\mathrm{FF}}\left(X\right)$ be the set of Fermatean fuzzy ideals of X then for $\delta_{F}\;\in \;I_{\mathrm{FF}}\left(X\right)$ for $\delta_{F}\;\&\;\theta_{F}\;\in \;I_{\mathrm{FF}}\left(X\right)\;$ we have:

1.
$U\left(\delta_{F},t\right)=\left\{x\;\in \;X:\delta_{F}\left(x\right)\;\geq \;t,t\;\in \;\left[0,1\right]\right\}$

2.
$L\left(\theta_{F},k\right)=\left\{x\;\in \;X:\theta_{F}\left(x\right)\;\leq \;k,k\;\in \;\left[0,1\right]\right\}$ are called Fermatean level ideal of X and $U\left(\delta_{F},t\right)$ and $L\left(\theta_{F},k\right)$ are called upper level ideal and lower-level ideal respectively.

Theorem 3.15: Let X be a BN-algebra and $\delta_{F},\theta_{F}\;\in \;I_{\mathrm{FF}}\left(X\right),\theta_{F}\left(x\right)=1-\delta_{F}\left(x\right)$ if and only if the non-empty level subsets $U\left(\delta_{F},t\right)$ and $L\left(\theta_{F},k\right),t,k\;\in \;\left[0,1\right]\;$ are ideals of X .

Proof: Let $\delta_{F},\theta_{F}\;\;\in \;I_{\mathrm{FF}}\left(X\right)$ and t,k∈[0,1] with $\theta_{F}\left(x\right)=1-\delta_{F}\left(x\right).$ We need to prove $U\left(\delta_{F},t\right)$ and $L\left(\theta_{F},k\right)$ are ideals of X.

Let $x_{0}\;\in \;X\;$ such that $\;\delta_{F}\left(x_{0}\right)\;\geq \;t,t\;\in \;\left[0,1\right]$ since $\delta_{F},\theta_{F}\;\in \;I_{\mathrm{FF}}\left(X\right)$ we have $\delta_{F}\left(0\right)\;\geq \;\delta_{F}\left(x_{0}\right)\;\geq \;t$ implies $\;0\;\in \;U\left(\delta_{F},t\right)$ .

Again, for $x_{0}\;\in \;$ x, such that $\theta_{F}\left(x_{0}\right)\;\leq \;k$ and $\theta_{F}\left(0\right)\;\leq \;\theta_{F}\left(x_{0}\right)\;\leq \;k$ it follows that $\theta_{F}\left(0\right)\;\leq \;k$ hence $0\;\in \;L\left(\theta_{F},k\right).$

Let x,y∈X and $\;x\;*\;y,y\;\in \;U\left(\delta_{F},t\right),t\;\in \;\left[0,1\right]$ , imply $\delta_{F}\left(x\;*\;y\right)\;\geq \;t\;$ and $\delta_{F}\left(y\right)\;\geq \;t$ since $\delta_{F}\;\in \;I_{\mathrm{FF}}\left(X\right),\delta_{F}\left(x\right)\;\geq \;\delta_{F}\left(x\;*\;y\right)\;\wedge \;\delta_{F}\left(y\right)\;\geq \;t.\;\delta_{F}\left(x\right)\;\geq \;t$ it follows that $x\;\in \;U\left(\delta_{F},t\right).$

Again let x,y∈X such that $x\;*\;y,y\;\in \;L\left(\theta_{F},k\right)$ then we have $\theta_{F}\left(x\;*\;y\right)\;\leq \;k$ and $\theta_{F}\left(y\right)\;\leq \;k$ since $0\;\leq \;{\left(\theta_{F}\left(x\right)\right)}^{3}+{\left(\delta_{F}\left(x\right)\right)}^{3}\;\leq \;1$ for all x∈X and $\theta_{F}\;\in \;I_{\mathrm{FF}}\left(X\right)$ we get $\theta_{F}\left(x\right)\;\leq \;\theta_{F}\left(x\;*\;y\right)\;\vee \;\theta_{F}\left(y\right)\;\leq \;k$ it follows that $\theta_{F}\left(x\right)\;\leq \;k$ hence $x\;\in \;L\left(\theta_{F},k\right)$ therefore $U\left(\delta_{F},t\right)\;$ and $L\left(\theta_{F},k\right),t,k\;\in \;\left[0,1\right]$ are ideals of X.

Conversely, suppose $U\left(\delta_{F},t\right)$ and $\;L\left(\theta_{F},k\right),t,k\;\in \;\left[0,1\right]$ are ideals of x we must prove $I_{\mathrm{FF}}\left(X\right)$ is Fermatean fuzzy ideal of X for each $t\;\in \;\left[0,1\right],U\left(\delta_{F},t\right)$ is non-empty if $x_{0}\;\in \;X$ such that $\delta_{F}\left(0\right)\delta_{F}\left(x_{0}\right)=s\;\in \;\left[0,1\right]$ then $U\left(\delta_{F},s\right)\neq \emptyset$ by assumption $U\left(\delta_{F},s\right)$ is an ideal of *x* hence $0\;\in \;U\left(\delta_{F},s\right)$ so that $\delta_{F}\left(0\right)\;\geq \;s$ which is a contradiction hence $\delta_{F}\left(0\right)\;\geq \;\delta \_F\left(x\_0\right)\;\geq \;s\;\in \;\left[0,1\right],x_{0}\;\in \;X.$

Let x,y∈X , such that $\delta_{F}\left(x\right)<\delta_{F}\left(x\;*\;y\right)\;\wedge \;\delta_{F}\left(y\right).\;\mathrm{Let}\;\beta =½\left\{\delta_{F}\left(x\right)+\left(\delta_{F}\left(x\;*\;y\right)\;\wedge \;\delta_{F}\left(y\right)\right)\right\}\;$ such that $\delta_{F}\left(x\right)<\beta <\delta_{F}\left(x\;*\;y\right)\;\wedge \;\delta_{F}\left(y\right)\;\leq \;\delta_{F}\left(x\;*\;y\right)\;$ and β < δ_f(y) hence $x\;*\;y,y\;\in \;U\left(\delta_{F},\beta \right).$ But $x\;\notin \;U\left(\delta_{F},\beta \right)$ this is impossible as $U\left(\delta_{F},t\right)\;$ is an ideal of X to show $\theta_{F}$ is a Fermatean fuzzy ideal is simply taking the reverse of the proof done for $\delta_{F}$ . This completes the proof.

## 4. Conclusions

This paper has addressed a significant gap in the study of algebraic structures by exploring the application of Fermatean fuzzy sets to the ideals and sub-algebras of BN-algebras. The findings confirm that Fermatean fuzzy sets enrich the understanding of BN-algebras by accommodating imprecision more effectively, establishing new structural properties, and broadening the scope of algebraic studies under uncertainty. This makes them a valuable tool not only for theoretical mathematics but also for applications in computer science, decision-making systems, and information processing. For the future this work can be extended to other algebraic structures like BCK-algebra, BCCI-algebra, BCC-algebra, BG-algebra and related. It can also be applied to deal with more complex problems.

## Data Availability

No datasets were generated or analyzed during this study. All results are derived analytically, and all supporting information is fully contained within the manuscript.
