# Supplementary material for: Some Results of Fermatean Fuzzy Set on Subalgebras and Ideals of Bn-Algebras
Source: F1000Res. 2026 May 7;15:163. Originally published 2026 Feb 2. [Version 2] doi: 10.12688/f1000research.175837.2 (PMC13213361; doi:10.12688/f1000research.175837.2)
Supplement: Peer review history

# Reviewer report: Reviewer response for version 2

**DOI:** 10.5256/f1000research.198517.r483261

**Published:** 2026-07-27

**Authors:** Prakasam Muralikrishna

**Affiliations:** 1 Muthurangam Government Arts College, Vellore, India

**Comments**

- In this paper, the authors applied Fermatean fuzzy sets to the ideals and sub-algebras of BN-algebras.
- The manuscript is well written, well presented and contains good results.
- The work is interesting and they also validated the concept with a suitable example. 
- It will help a lot to researchers working in the related field.

But few areas to be improved little more.

 1. Write more about motivation and applications.

 2. Remove trivial and known preliminaries.

 3. Since it is full of theoretical development based on one particular algebraic structure, it can be compared with other related structures and may give some counter examples whenever needed.

 4. Conclusion may be strengthened.

 5. Cite few more recent references.

**Overall , the article can be accepted based on the suggestions given above and rewrite the points revised majorly.**

Is the work clearly and accurately presented and does it cite the current literature?

Yes

If applicable, is the statistical analysis and its interpretation appropriate?

No

Are all the source data underlying the results available to ensure full reproducibility?

Partly

Is the study design appropriate and is the work technically sound?

Partly

Are the conclusions drawn adequately supported by the results?

Partly

Are sufficient details of methods and analysis provided to allow replication by others?

Partly

Reviewer Expertise:

Fuzzy Algebra

I confirm that I have read this submission and believe that I have an appropriate level of expertise to confirm that it is of an acceptable scientific standard, however I have significant reservations, as outlined above.

# Reviewer report: Reviewer response for version 2

**DOI:** 10.5256/f1000research.198517.r482408

**Published:** 2026-05-26

**Authors:** Kalavath Anjaneyulu Naik

**Affiliations:** 1 Acharya Nagarjuna University, Guntur, Andhrapradesh, India

Title: Some Results of Fermatean Fuzzy Set on Subalgebras and Ideals of Bn-Algebras

 I have reassessed the manuscript after the revisions. The authors have addressed the concerns I raised regarding redundancies, references, and clarity of the conclusion. The removal of Definition 2.11, correction of Definition 2.4, and the updated conclusion adequately resolve my previous reservations.

 I am now happy to change my status to **Approved**.

Is the work clearly and accurately presented and does it cite the current literature?

Yes

If applicable, is the statistical analysis and its interpretation appropriate?

I cannot comment. A qualified statistician is required.

Are all the source data underlying the results available to ensure full reproducibility?

No source data required

Is the study design appropriate and is the work technically sound?

Yes

Are the conclusions drawn adequately supported by the results?

Partly

Are sufficient details of methods and analysis provided to allow replication by others?

Partly

Reviewer Expertise:

Fuzzy Algebraic Structures, Hyper Algebraic Structures, Intuitionistic Fuzzy Algebraic Structures, Neutrosophic Fuzzy Algebraic Structures, Soft Sets, Rough Sets and Their Decision-Making Systems in Different algebraic structures.

I confirm that I have read this submission and believe that I have an appropriate level of expertise to confirm that it is of an acceptable scientific standard.

# Reviewer report: Reviewer response for version 1

**DOI:** 10.5256/f1000research.193852.r472221

**Published:** 2026-04-03

**Authors:** Jehad R. Kider

**Affiliations:** 1 University of Technology, Baghdad, Iraq

Thank you for sending me to review this paper.

 1.The main question addressed by this research, is can we state and prove some theorems discussed in the fermatean fuzzy set on the ideals of BN-algebras and applications. we also can we extend the notions of an ideal and a normal ideal in a fermatean fuzzy set on the ideals of Bn-algebras.

 2.Yes, this research is in the relevant to the field as well as it addresses a specific gap in the field because it is shown that fermatean fuzzy level subalgebras and fermatean fuzzy level ideals correspond to classical subalgebras and ideals of Bn-algebras. several characterizations and closure properties are established, supported by illustrative examples and rigorous proofs.

 3. In this research the authors results demonstrate that fermatean fuzzy sets significantly enrich the theory of Bn-algebras by accommodating higher degrees of uncertainty.

 4. Generally, the paper is well written and has a good structureas well as the theoretical framework is built systematically, starting from definitions to illustrative examples and theorems so that I do not suggest any specific improvements should the authors consider regarding the methodology.

 5. The conclusions are consistent with the evidence and the arguments presented as well as they address the main question posed. The results presented in this paper

  provides a solid theoretical foundation for further studies and potential applications in algebraic logic and uncertainty-based systems.

 6. The references are appropriate.

 7.The research contains tables which are good but no figures.

 Taking the above into consideration, I recommend the paper for indexing in your journal.

Is the work clearly and accurately presented and does it cite the current literature?

Yes

If applicable, is the statistical analysis and its interpretation appropriate?

I cannot comment. A qualified statistician is required.

Are all the source data underlying the results available to ensure full reproducibility?

No source data required

Is the study design appropriate and is the work technically sound?

Yes

Are the conclusions drawn adequately supported by the results?

Yes

Are sufficient details of methods and analysis provided to allow replication by others?

Yes

Reviewer Expertise:

Fuzzy Metric Spaces, Fuzzy Normed Spaces, Fuzzy Inner Product Spaces.

I confirm that I have read this submission and believe that I have an appropriate level of expertise to confirm that it is of an acceptable scientific standard.

# Reviewer report: Reviewer response for version 1

**DOI:** 10.5256/f1000research.193852.r459976

**Published:** 2026-03-09

**Authors:** Kalavath Anjaneyulu Naik

**Affiliations:** 1 Acharya Nagarjuna University, Guntur, Andhrapradesh, India

**Review Recommendations:**

1. **Clarity and Organization:** The paper is easy to follow, with a clear abstract, background, methods, results, and conclusions. But some sections could use more examples to help readers less familiar with fermatean fuzzy sets and bn-algebras.
2. **Technical Depth and Novelty:** The study adds a lot by to bn-algebras by using fermatean fuzzy sets, which handles higher degrees of uncertainty. It new to apply fermatean fuzzy sets to bn-algebras, and it could be really useful for dealing with complex uncertainty in information theory.
3. ** Methodology:** The method involves defining and looking at fermatean fuzzy subalgebras and ideals in bn-algebras using algebraic techniques. It’s thorough and supported by illustrative examples and proofs. Linking the definitions and theorems to existing research would make the method stronger.
4. **Results and Implications:** The results show that fermatean fuzzy sets add a lot to bn-algebras. This is big for areas like algebraic logic and systems that deals with uncertainty, and could be used in computer science, decision-making, and information processing.
5. **Future Work:** The authors say more research could be done in areas like algebraic logic and systems that deal with uncertainty. If the authors added more details, it would give other clear path to follow for future research, like exploring new uses or digging deeper into theory.
6. ** References and Citations:** The paper mentions key research (like Zadeh, Atanassov, Senapati et al., Komori, Neggers, H.S.Kim, Yager). Checking these references are right and complete is important. Also, talking about how this work fits into what’s happening in research now could make it more impactful.  
7. **Accessibility:** The paper is technically wise but making it easier for more people to understand (like graduate students or researchers from other fields) would be good. They could add examples at the start or a list of key terms like “fermatean fuzzy set,” “bn-algebra,” and “level cut.”
8. Definitions 2.1, 2.4, and 2.11 seem to be similar or identical. Please check for typographical errors or clarify the distinction between these definitions if they're meant to be different.
9. References 6 and 7 appear to be duplicates; reference 7 is the correct one. Please remove or correct reference 6.

**Potential Areas for Improvement:**

-
  - Enhance clarity with additional illustrative examples for complex concepts.
  - Discuss limitations of the current study.
  - Suggest specific future research directions beyond general applications.
  - Ensure all technical terms are well-defined for a general readership.

Is the work clearly and accurately presented and does it cite the current literature?

Yes

If applicable, is the statistical analysis and its interpretation appropriate?

I cannot comment. A qualified statistician is required.

Are all the source data underlying the results available to ensure full reproducibility?

No source data required

Is the study design appropriate and is the work technically sound?

Yes

Are the conclusions drawn adequately supported by the results?

Partly

Are sufficient details of methods and analysis provided to allow replication by others?

Partly

Reviewer Expertise:

Fuzzy Algebraic Structures, Hyper Algebraic Structures, Intuitionistic Fuzzy Algebraic Structures, Neutrosophic Fuzzy Algebraic Structures, Soft Sets, Rough Sets and Their Decision-Making Systems in Different algebraic structures.

I confirm that I have read this submission and believe that I have an appropriate level of expertise to confirm that it is of an acceptable scientific standard, however I have significant reservations, as outlined above.

# Response

**Published:** 2026-03-12

**Authors:** Gerima Tefera

**Affiliations:** Mathematics, Wollo University, Dessie, Amhara, Ethiopia

Response for comment"

 This article has many diversified Applications in related area of uncertainty.  In addition, this research can be extended to BCK-algebra, BGG-algebra, BG-algebra, KU-algebra and other related algebraic structures.

# Response

**Published:** 2026-04-06

**Authors:** Derebew Derso

**Affiliations:** Mathematics, Woldia University, Weldiya, Amhara, Ethiopia

Author Response to Reviewer Comments

**Reviewer:** Anjaneyulu Naik Kalavath

 1. Regarding Future Work

**Author’s Response:** We appreciate the reviewer's suggestion. Fermatean Fuzzy Sets (FFS) offer a robust framework for handling uncertainty in decision-making. We have updated the conclusion to highlight that the proposed concepts of **Fermatean BN-subalgebras** and **Fermatean ideals** can be extended to other algebraic structures, including **B-algebras, BCK/BCC-algebras, and BG-algebras**, as well as other related mathematical frameworks.

 2. References and Citations

**Author’s Response:** We thank the reviewer for the careful check of our references. We have re-evaluated references [6] and [7]. In accordance with the reviewer’s suggestion, **reference [6] has been removed**, and the citations in the main text have been adjusted accordingly to ensure accuracy.

 3. Accessibility and Preliminaries

**Author’s Response:** This paper presents original research on Fermatean Fuzzy BN-algebras. To maintain a concise focus on our new contributions, we have assumed the reader possesses a foundational understanding of BN-algebras and Fermatean Fuzzy Set theory. However, to ensure the paper remains self-contained, we have clearly stated the essential definitions of BN-algebras and FFS in the **Preliminaries section**. We believe this provides sufficient context for the reader without redundant elaboration.

 4. Revisions to Definitions (2.1, 2.4, and 2.11)

**Author’s Response:** We agree with the reviewer’s observation regarding the redundancy in these definitions. These errors occurred during the final editing stage. We have made the following corrections:

- **Definition 2.1:** Replaced with a more fundamental definition of Fermatean Fuzzy Sets from reference [3].
- **Definition 2.11:** This definition has been **removed** to avoid redundancy. We thank the reviewer for their keen eye and for helping us improve the technical rigor of the manuscript.

5. Potential Areas for Improvement and Limitations

**Author’s Response:** We have addressed the study's limitations in the revised manuscript. Specifically:

- **Literature Gap:** We acknowledge that there is currently limited literature regarding the application of Fermatean Fuzzy Sets specifically within algebraic structures, which constrained our comparative analysis.
- **Future Scope:** Because FFS can model more complex uncertainties than standard Intuitionistic Fuzzy Sets, we have suggested that future research should focus on its application in **algebraic logic, decision theory, and control theory.** ---
